# Real-World Performance of the EasyPGX^®^ Ready Epidermal Growth Factor Receptor Assay for Genomic Testing of Non-Small Cell Lung Cancer Samples

**DOI:** 10.3390/biomedicines13040814

**Published:** 2025-03-28

**Authors:** Michael Bento Schmid, Izadora Demmer, Sandra Floriani, Diana Born, Wolfram Jochum

**Affiliations:** 1 Institute of Pathology, Cantonal Hospital St. Gallen, 9007 St. Gallen, Switzerland; michael.schmid@h-och.ch (M.B.S.); izadora.demmerbuchs@h-och.ch (I.D.);; 2 University of Zurich (UZH), 8006 Zurich, Switzerland

**Keywords:** EGFR variant testing, non-small cell lung cancer, RT-qPCR, NGS

## Abstract

**Background/Objectives**: Activating epidermal growth factor receptor (*EGFR*) variants is the most common targetable alteration in non-small cell lung cancer (NSCLC). Clinical decision-making requires fast and reliable detection of *EGFR* variants in early and advanced NSCLC, but limited available tissue necessitates tissue-sparing approaches and optimized sample management. The objective of this study was to assess the performance of the commercial EasyPGX^®^ ready EGFR assay using real-world clinical NSCLC samples. **Methods**: A consecutive cohort of 804 non-squamous NSCLC samples was prospectively analyzed with the real-time quantitative polymerase chain reaction (RT-qPCR)-based EasyPGX^®^ ready EGFR assay (Diatech Pharmacogenetics, Jesi, Ancona, Italy) and compared to next-generation sequencing (NGS) assays. **Results**: NGS revealed conclusive results in 99.7% samples, of which 11.1% had at least one *EGFR* variant. The most common variants were exon 19 deletions and p.L858R. The RT-qPCR-based assay identified *EGFR* variants with high accuracy (overall concordance rate 94.3%) over a broad range of clinical sample types, variant allele frequencies, tumor cell contents and deoxyribonucleic acid (DNA) input amounts. **Conclusions**: This study demonstrates that the EasyPGX^®^ ready EGFR assay is a valid approach for the rapid detection of common *EGFR* variants in real-world clinical NSCLC samples with DNA inputs as low as 5 ng (less than the 15 ng recommended by the manufacturer), improving sample management in small specimens with limited quantity of nucleic acids.

## 1. Introduction

Activating epidermal growth factor receptor (*EGFR*) variants are the second most common oncogenic driver event in non-small cell lung cancer (NSCLC) and remain the most common targetable alteration [1,2]. They occur mainly in the adenocarcinoma subtype and their incidence is highly dependent on the tested population (12–47%) [3]. The most frequent *EGFR* variants are exon 19 deletions and the p.L858R missense variant in exon 21, which together comprise 85–90% of all *EGFR* variants [4]. Uncommon *EGFR* variants include exon 18 variants (mainly p.G719X), exon 20 insertions, exon 20 p.S768I, and exon 21 p.L861Q.

Standard-of-care for advanced NSCLC with sensitizing *EGFR* variants (e.g., exon 19 deletions, p.L8585R) are *EGFR* tyrosine kinase inhibitors (TKI) of the first (e.g., erlotinib, gefitinib), second (e.g., afatinib) and third generation (e.g., osimertinib) [5]. In addition, detection of acquired resistance variants (e.g., p.T790M, MET proto-oncogene [*MET*] amplification) allows adapting the treatment at the time of progression. Therefore, international consensus statements recommend broad molecular testing using next generation sequencing (NGS) rather than single-gene *EGFR* testing in advanced disease [6].

More recently, the use of *EGFR* TKI and therefore *EGFR* variant testing has begun to shift from the advanced to the early, adjuvant [7,8] and neoadjuvant [9,10] setting. Therefore, selective *EGFR* testing may be more appropriate to guide treatment decisions in early NSCLC, in contrast to broad molecular profiling in advanced NSCLC. For this purpose, the high sensitivity of NGS may not be essential and advantages of selective *EGFR* testing such as short assay turnaround time (TAT) and lower costs may gain importance.

Testing for somatic *EGFR* variants can be performed by a broad range of methods, including Sanger sequencing, real-time quantitative PCR (RT-qPCR), amplification refractory mutation system PCR (ARMS-PCR), NGS and digital PCR. Suitable specimens include formalin-fixed paraffin-embedded (FFPE) histologic material (biopsies, resections) or cytological material (FFPE cell blocks, non-FFPE samples such as smears and touch preparations) [11]. However, limited quantity of tissue owing to minimally invasive tissue biopsies in advanced disease remains a key challenge and requires the adoption of tissue-sparing approaches and optimized sample management in biomarker testing of advanced NSCLC [11]. While NGS is more cost-effective than single-gene testing for multiple targets [12], it has a relatively long average TAT of around 10–14 days. Alternative analytical techniques, such as immunohistochemistry (IHC), fluorescence in situ hybridization (FISH) and RT-qPCR have reduced TAT and are therefore commonly integrated in customized fast track panels to screen for certain biomarkers in NSCLC (e.g., IHC as a surrogate marker for anaplastic lymphoma kinase [*ALK*] fusions, FISH for *ALK* and ROS proto-oncogene 1 [*ROS1*] fusions, RT-qPCR for *EGFR* variants) [12]. In addition, biomarker testing for programmed cell death ligand 1 (PD-L1) is mandatory in advanced NSCLC and can only be performed by IHC [12]. An economical use of the available tissue is thus paramount, in particular in view of the rapidly evolving landscape of multimodal biomarker testing in NSCLC [13].

The EasyPGX^®^ ready EGFR assay (Diatech Pharmacogenetics, Jesi, Ancona, Italy) is a CE-IVD-marked allele-specific RT-qPCR test designed to detect clinically relevant *EGFR* variants in exons 18 through 21 including deletions in exon 19 and insertions in exon 20, allowing for shorter TAT and lower costs compared to NGS. The aim of the current study was to evaluate the diagnostic performance of the EasyPGX^®^ ready EGFR assay using a prospective cohort of real-world clinical NSCLC samples. Specifically, we analyzed the assay’s performance in samples of low quantity and/or quality, its accuracy compared to NGS-based testing, and the feasibility of its integration into a customized in-house fast track testing approach for NSCLC.

## 2. Materials and Methods

### 2.1. Technical Verification

For local verification of the EasyPGX^®^ ready EGFR assay, we used the 5% Multiplex I cfDNA Reference Standard (Horizon Discovery, Waterbeach, Cambridge, UK) that contains DNA with five clinically relevant *EGFR* variants (p.G719S, p.E746_A750del, p.M766_A767insASV, p.T790M, p.L858R) at variant allelic frequencies (VAF) of 5% (20% for p.G719S). All five variants are detectable by the EasyPGX^®^ ready EGFR assay. We then retrospectively searched the laboratory information management system (LIMS) of the Institute of Pathology, Cantonal Hospital St. Gallen, for NSCLC samples on which NGS-based *EGFR* variant testing had been performed and for which remaining extracted DNA was available.

### 2.2. Performance Evaluation Cohort

The cohort included consecutive non-squamous NSCLC samples referred to the Institute of Pathology, Cantonal Hospital St. Gallen for molecular testing between June 2020 and November 2024. The cohort comprised carcinomas with morphological features of adenocarcinoma and undifferentiated carcinomas with or without TTF1 immunopositivity. Squamous cell carcinomas based on morphological features or immunopositivity for p40 or p63 were excluded. Patient-related data (age at diagnosis, gender), sample characteristics (sample type, tumor cell content) and *EGFR* testing-related data (DNA input, sequence variants, VAF, TAT) were prospectively collected during genomic testing. The clinico-pathologic and genomic characteristics of the performance evaluation cohort are summarized in Table 1.

### 2.3. Specimen Preparation and Pathological Analysis

NSCLC biopsy and resection specimens were fixed in neutral buffered formalin (4%) and processed for histological analysis using standard methods. Cell blocks were prepared from cytological specimens (cytological smears and liquid-based preparations). By centrifugation at 2000 rpm for 10 min, followed by embedding the cell pellets in 10% AGAR, fixation in neutral buffered formalin (4%), and paraffin embedding. Sections (2 μm thick) were stained with hematoxylin and eosin (H&E). Cytological specimens were prepared using standard methods and Papanicolaou-stained on a TST 44 or TST 44C multi-stainer (Medite Service AG, Dietikon, Zurich, Switzerland). Carcinomas were classified according to the World Health Organization (WHO) Classification of Thoracic Tumors (2021).

### 2.4. Nucleic Acid Extraction

H&E-stained sections and Papanicolaou-stained slides were used to evaluate tumor cell content and to mark tumor regions or cells. A minimum of 20% tumor cell content was required for NSCLC samples to be eligible for molecular testing. Genomic DNA was prepared from unstained 3 μm thick sections of FFPE tissue blocks or cell blocks after manual microdissection of tumor cells. A new blade was used for each sample to prevent cross-contamination. Alternatively, cells were scraped from stained cytological specimens and processed without destaining. For both FFPE and non-FFPE material, DNA was extracted using the Maxwell^®^ CSC DNA FFPE Kit or Maxwell^®^ RSC DNA FFPE Kit (Promega, Fitchburg, WI, USA). The extraction process was automated using the Maxwell^®^ CSC or Maxwell^®^ RSC Instruments, set up according to the manufacturer’s instructions (Promega, Fitchburg, WI, USA). Protocols were adapted in-house for optimal DNA yield and purity. DNA was eluted in nuclease-free water, and concentrations were measured using the Qubit™ dsDNA Quantification Assay Kit and Qubit 2.0 Fluorometer instrument (Thermo Fisher Scientific, Waltham, MA, USA). DNA samples were stored at 4–8 °C for up to one week or at −20 °C for long-term storage until analysis.

### 2.5. Next Generation Sequencing

NGS of NSCLC DNA was performed with the Oncomine™ Comprehensive Assay v3 or the Oncomine™ Focus Assay on the Ion GeneStudio™ S5 System (Thermo Fisher Scientific, Waltham, MA, USA) following the manufacturer’s instructions. Both assays allow for the detection of *EGFR* single-nucleotide variants, small insertions, small deletions and copy number gains. The Torrent Suite™ software (versions 5.12, 5.16 and 5.18; Thermo Fisher Scientific, Waltham, MA, USA) was used to visualize and perform the initial quality control (chip loading density, median read length, number of mapped reads, mean depth). Variant calling was performed with the Ion Reporter^®^ analysis software (versions 5.12, 5.16 and 5.20) using the assay-specific workflows.

### 2.6. EasyPGX^®^ Ready EGFR Assay

The EasyPGX^®^ ready EGFR assay is composed of an internal control assay for assessment of sample DNA content specifically on the *EGFR* gene, and seven assays for the qualitative detection of the *EGFR* variants p.G719X (exon 18), exon 19 deletions, p.T790M and p.S768I (exon 20), exon 20 insertions as well as p.L858R and p.L861Q (exon 21). The assay selectively amplifies altered *EGFR* sequences in samples that contain altered and wild-type DNA. All analyses were performed on the EasyPGX^®^ qPCR instrument 96 according to the manufacturer’s instructions (Diatech Pharmacogenetics, Jesi, Ancona, Italy), with the EasyPGX^®^ ready EGFR kit version 2018/06 or 2022/05. Raw data were analyzed with EasyPGX^®^ Analysis software version 4.0.1 or 4.0.14 according to the manufacturer’s instructions (Diatech Pharmacogenetics, Jesi, Ancona, Italy). For FFPE samples, the manufacturer recommends using 15 ng of sample DNA for each reaction tube. For samples of limited quantity, however, this input requirement proved unfeasibly high, as in these cases, the residual material was not sufficient to perform further molecular testing ordered by the clinicians (FISH for *ALK* and *ROS1*, DNA- and RNA-based NGS). Therefore, variable DNA input ranging from 1–30 ng per reaction were tested by dispensing different DNA input volumes into the appropriate wells. Samples were categorized according to DNA input and assigned to one of three categories as follows: low (<5 ng), intermediate (5–15 ng) and high (≥15 ng).

### 2.7. Turnaround Time

TAT was defined as the interval between registration of the test order in the LIMS and release of the molecular pathology report.

### 2.8. Statistical Analysis

Data were analyzed with descriptive statistics using IBM SPSS Statistics software version 27.0 (IBM Corp., Armonk, NY, USA) and GraphPad Prism software version 7 (GraphPad Software Inc., San Diego, CA, USA). The results are presented as frequencies for categorical variables and as mean ± standard deviation and range for continuous variables. To evaluate the diagnostic performance of the EasyPGX^®^ ready EGFR assay, concordance values were calculated using NGS-based *EGFR* test results as the reference. Clinically relevant *EGFR* variants included common and uncommon *EGFR* alterations: p.G719X (exon 18), exon 19 deletions, exon 20 insertions, p. T790M (exon 20), p.L858R (exon 21), and p.L861Q (exon 21).

## 3. Results

### 3.1. Verification of the EasyPGX^®^ Ready EGFR Assay

When using 3 or 2 ng reference material DNA input, the EasyPGX^®^ ready EGFR assay detected all *EGFR* variants present in the reference material. DNA input of 1 ng, however, was not sufficient to obtain conclusive test results (Table S1). Subsequently, eight NSCLC DNA samples obtained from various sample types (biopsy, resection specimen, cytological cell block, cytological specimens) were analyzed. For DNA inputs ≥15 ng, as recommended for FFPE samples by the manufacturer, conclusive and concordant results were obtained in all cases (samples 1–8 in Table S2). The verification was then extended to 12 additional clinical NSCLC samples with DNA inputs <15 ng (samples 9–20 in Table S2). In addition, sample 8 from the initial verification was retested with various DNA inputs <15 ng. Conclusive and concordant results were obtained for all tests performed with DNA inputs <15 ng.

### 3.2. EGFR Variant Testing by NGS

In total, 804 non-squamous NSCLC samples were included in the clinical performance cohort. Mean patient age was 69.6 years (standard deviation 10.1 years, range 28–98 years), with an equal gender distribution. Of the 804 samples, 487 were biopsies (60.6%), 193 were surgical resection samples (24.0%), 106 were cytological cell blocks (13.2%) and 18 were cytological specimens (cytological smears and liquid-based preparations; 2.2%). The clinico-pathologic and genomic characteristics of the cohort are summarized in Table 1.

NGS revealed conclusive results in 802 of 804 samples (99.7%), of which 89 (11.1%) had at least one *EGFR* variant (Table 1). Two of 804 NGS tests failed (test failure rate 0.3%), both occurring in the low (<5 ng) DNA input category. There were no failed tests in samples with DNA inputs ≥5 ng.

**Figure 1:**
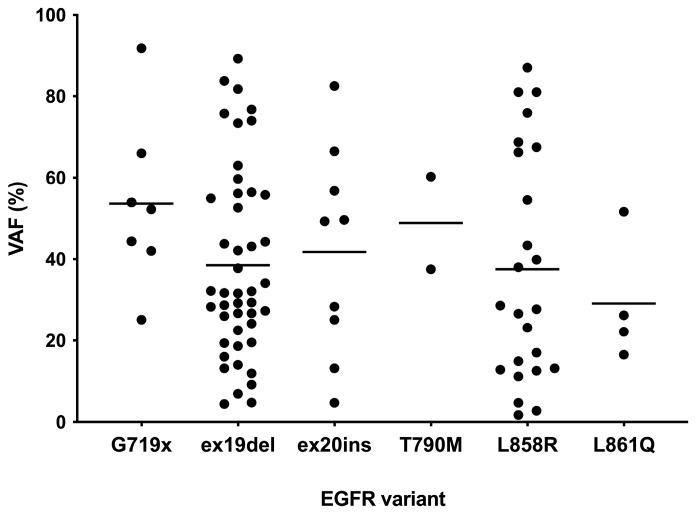
VAF distribution of *EGFR* variants detected by NGS in the clinical performance cohort. Horizontal lines depict mean VAF of each *EGFR* variant.

NGS detected 91 *EGFR* variants (Table 1). Of these, 45 (49.4%) were exon 19 deletions and 24 (26.4%) were the p.L858R variant. Less common *EGFR* variants were exon 20 insertions in 9.9% (n = 9), p.G719X in 7.7% (n = 7), p.L861Q in 4.4% (n = 4) and p.T790M in 2.2% (n = 2). Both p.T790M variants occurred in samples that also tested positive for p.L858R. *EGFR* variants were present at VAF ranging from 1.7% to 89.2% (Figure 1).

### 3.3. EGFR Variant Testing by the EasyPGX^®^ Ready EGFR Assay

All 804 non-squamous NSCLC samples of the cohort were analyzed with the EasyPGX^®^ ready EGFR assay as part of a customized in-house fast track testing approach for NSCLC. Based on the results of the technical verification, NSCLC samples with less than 15 ng DNA input were also tested. Valid results were obtained in 763 of 804 samples (94.9%). The EasyPGX^®^ ready EGFR assay was positive in 87 of 804 samples (10.8%). The most common *EGFR* alterations were exon 19 deletions in 50.6% (n = 44), followed by exon 21 p.L858R in 25.3% (n = 22), exon 18 p.G719X in 9.2% (n = 8), exon 20 insertions in 8.0% (n = 7), exon 21 p.L861Q in 4.6% (n = 4) and exon 20 p.T790M in 2.3% (n = 2). Of note, both p.T790M-positive cases also tested positive for a concurrent p.L858R variant.

### 3.4. Concordance Analysis

Performance was assessed as the degree of concordance between the *EGFR* variants detected by the EasyPGX^®^ ready EGFR assay and the variants identified by NGS (Table 2).

The overall concordance rate of test results was 94.3% (760/806). For the two most frequent *EGFR* variants, exon 19 deletions and the p.L858R variant, concordance rates were 97.8% and 100.0%, respectively. The EasyPGX^®^ ready EGFR assay reliably detected all *EGFR* variants, including exon 20 insertions, across a broad range of VAF, ranging from 1.7% (a FFPE biopsy with the exon 21 p.L858R) to 89.2% (a FFPE biopsy with the exon 19 deletion p.E746_A750del) (Figure 1).

### 3.5. Discordant Samples

In four out of nine samples, NGS detected *EGFR* exon 20 variants that were not detected by the EasyPGX^®^ ready EGFR assay (concordance rate 55.6%). These were two exon 20 duplications p.S768_D770dup (one with VAF 4.7%, cytological cell block, high DNA input category; the other with VAF 66.5%, FFPE resection, intermediate DNA input category), one exon 20 insertion p.N771_P772insG (VAF 56.8%, FFPE biopsy, high DNA input category) and one exon 20 deletion/insertion p.N771delinsGF (VAF 82.5%, FFPE biopsy, intermediate DNA input category). Discordant test results were due to the assay design, since both kit versions of the EasyPGX^®^ ready EGFR assay used in our study were not able to detect these *EGFR* exon 20 variants.

In one case where the EasyPGX^®^ ready EGFR assay yielded no valid result (low DNA input category), NGS was able to detect the classical exon 19 deletion p.E746_A750del (VAF 14.0%, FFPE biopsy).

Lastly, three cases that tested positive by the EasyPGX^®^ ready EGFR assay (two exon 20 insertions, one p.G719X variant) were negative on NGS testing and were regarded as false-positive results. The majority of discordant samples were FFPE samples, and all but one pertained to the intermediate or low DNA input category (Table 3).

### 3.6. Failure Rate

In our cohort, the overall failure rate of the EasyPGX^®^ ready EGFR assay was 5.1% (Table 4). A strong association between DNA input and failure rate was observed. Among the samples with high DNA input (>15 ng per reaction well), the success rate was 100% (valid results in 276/276 samples). The success rates for samples with intermediate (5–15 ng) and low (<5 ng) DNA input were 99.6% (valid results in 272/273 samples) and 84.3% (valid results in 215/255 samples), respectively. Almost all invalid tests (40/41, 97.6%) occurred in samples with a DNA input <5 ng. The single test failure in the intermediate input category had a DNA input of exactly 5 ng and was therefore right on the edge of the low-input category.

In addition, we observed an association between failure rates and sample type (Table 5). Cytological specimens had the highest failure rate of 16.7% (3/18 tests failed), followed by cytological cell blocks with 8.4% (9/106 tests failed) and FFPE biopsies with 5.7% (28/487 tests failed). The lowest failure rate of 0.5% was observed in FFPE resection specimens (1/193 tests failed).

Lastly, we observed no association between test failure rate and initial tumor cell content of the sample determined by the pathologist. Samples with low initial tumor cell content of 20–30% (samples with <20% tumor cell content were excluded from molecular testing) had a test failure rate of 1.4% (one of 70 samples failed), compared to a test failure rate of 5.4% in samples with ≥40% tumor cell content (40 of 733 samples failed, one sample had no available tumor cell content).

### 3.7. Turnaround Times

**Figure 2:**
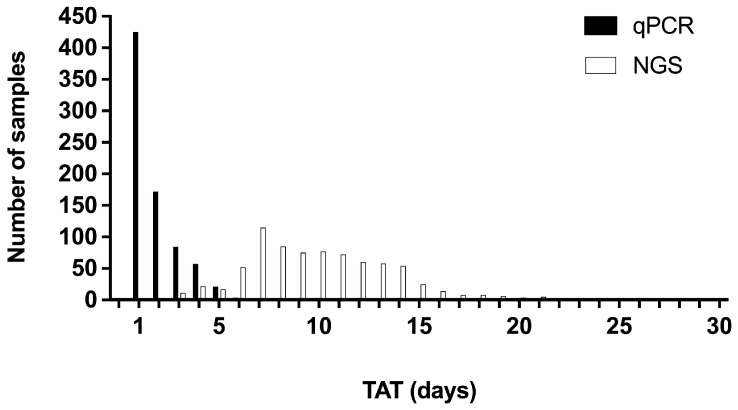
TAT distribution for the EasyPGX^®^ ready EGFR assay (qPCR) and NGS-based *EGFR* testing (NGS).

TATs were available for 775 of 804 samples (96.4%). TATs ranged from 1 to 12 days (mean 1.9 days, standard deviation 1.4 days) for the EasyPGX^®^ ready EGFR assay and from 2 to 30 days (mean 10.1 days, standard deviation 3.7 days) for NGS-based testing, respectively (Figure 2). With the EasyPGX^®^ ready EGFR assay, more than 50% of results were available to the clinician within one day after the LIMS registration of the test order, and more than 90% within the following four days. For NGS, it took 10 days to release more than 50% and 14 days to release more than 90% of the results, respectively.

## 4. Discussion

The objective of this study was to assess the performance of the commercial allele-specific RT-qPCR-based EasyPGX^®^ ready EGFR assay for the detection of *EGFR* variants using real-world clinical NSCLC samples in a routine diagnostic setting (tertiary hospital in Switzerland). We assessed a large cohort of NSCLC samples and found that the assay provides reliable test results for the most common *EGFR* variants across a broad range of clinical sample types, VAF and DNA input amounts. NGS was used as a reference method, which has demonstrated high sensitivity and specificity to detect *EGFR* variants [14].

In the present study, NGS identified *EGFR* variants in 11.1% of non-squamous NSCLC samples, while the most common *EGFR* alterations were exon 19 deletions (49.4%) and exon 21 p.L8585R (26.4%), followed by exon 20 insertions (9.9%), exon 18 p.G719X (7.7%), exon 21 p.L861Q (4.4%) and exon 20 p.T790M (2.2%). The observed overall rate of *EGFR* alterations agrees well with the frequency of *EGFR* variants in European (non-Asian) NSCLC patients, as do the frequencies of the two most common *EGFR* alterations (exon 19 deletions, p.L858R), together accounting for 75.8% in the present study [15]. To our knowledge, this study is the first to assess the prevalence of *EGFR* alterations in a large prospective cohort of non-squamous NSCLC patients in Switzerland.

More importantly, our results indicate that the assay provides reliable results for a broad range of DNA input amounts. The test success rate for the high (>15 ng) and intermediate (5–15 ng) DNA input category was 100% and 99.6%, respectively, while the single test failure in the intermediate DNA input category occurred in a sample with exactly 5 ng of input DNA, positioning it right on the edge to the low (<5 ng) DNA input category. Almost all invalid tests (97.6%) occurred in the low DNA input category. It is worth mentioning that within this category, failed tests were overrepresented in samples where DNA concentration was not measurable (failure rate 40%) when compared to samples with low (<5 ng) but measurable DNA input (failure rate 15.2%). The manufacturer recommends a DNA input of 15 ng sample DNA for each reaction tube, a prerequisite that in some settings is not feasible for small biopsies with limited material (i.e., customized in-house fast track panels where qPCR-based *EGFR* testing is followed by reflex NGS testing). The present findings indicate that the EasyPGX^®^ ready EGFR assay can provide reliable results even in samples of limited quantity with success rates approaching 100% if DNA inputs of >5 ng are used. The fact that 5–15 ng (as opposed to the >15 ng recommended by the manufacturer) of input DNA is sufficient represents the most important finding of this study and has the potential to improve sample management in small specimens with limited quantity of nucleic acids.

The present results further indicate that the EasyPGX^®^ ready EGFR assay provides reliable results for a broad range of clinical sample types and VAF. The assay reliably detected *EGFR* alterations over a VAF range from 1.7 to 89.2%, and the failure rate of samples with low tumor cell content (20–30%) was not higher than for samples with higher tumor cell content (≥40%). Performance of the EasyPGX^®^ ready EGFR assay was comparable between sample types, yielding reliable results for cytological specimens and cytological cell blocks, although with a higher test failure rate (16.7% and 8.4%, respectively) than for FFPE biopsies and FFPE resection specimens (5.7% and 0.5%, respectively). These findings are in concordance with what has been reported for other allele-specific PCR assays applied for *EGFR* variant testing [16,17]. In advanced NSCLC, cytological samples (e.g., pleural effusions, fine needle aspirates) are routinely used for cancer diagnosis but may provide limited DNA amounts. Whereas molecular testing from cytological specimens is possible, the cell block technique processes cytological samples as paraffin blocks that are suitable for a broad range of ancillary tests (IHC, FISH, PCR-based testing) [18]. However, *EGFR* variant testing on cytological samples also has its limitations. It has been shown, for example, that the *EGFR* p.T790M variant that occurs during treatment with first- or second-generation *EGFR* TKI [19] can be missed by the Idylla™ EGFR Mutation Test, especially in cytological samples with a low amount of amplifiable DNA and a low allelic fraction of the p.T790M-altered allele [17,20]. Here, the EasyPGX^®^ ready EGFR assay was able to reliably detect all p.T790M variants present in the sample cohort.

Relative to NGS-based testing, RT-qPCR-based methodologies confer the distinct advantage of markedly reduced TAT. In the present study, the mean TAT of the EasyPGX^®^ ready EGFR assay was 1.9 days, which is comparable to TAT reported for the Idylla™ EGFR Mutation Test [21,22,23,24]. The mean TAT for NGS-based testing in the present study, on the other hand, was 10.1 days, which on average is 8.2 days longer than for the EasyPGX^®^ ready EGFR assay. A recent survey on real-world *EGFR* testing practices of European pathology laboratories revealed an average TAT of 7–10 days when combining all methods [25]. The present results fall well within this range as well as within the recommended TAT of 10 days for molecular testing in NSCLC [26].

RT-qPCR-based approaches for *EGFR* variant testing such as the EasyPGX^®^ ready EGFR assay have several limitations when compared to NGS-based testing. First, most RT-qPCR-based approaches detect only alterations in a single gene (e.g., *EGFR* as in the present study), and multiple assays have to be performed to identify all targetable alterations in NSCLC samples [27,28]. Second, RT-qPCR-based approaches can only detect pre-specified genomic alterations. In the present study, the EasyPGX^®^ ready EGFR assay detected only 5 of the 9 (55.6%) exon 20 insertions identified by NGS. This finding is consistent with recent work by Pisapia et al. [29], who retrospectively compared NGS results of a large series of NSCLC samples with the reference ranges of four RT-qPCR assays, including the EasyPGX^®^ ready EGFR assay. All four assays detected fewer variants than NGS-based testing. Consistent with the results of our study, the RT-qPCR-based assays reliably detected the most common *EGFR* alterations (exon 19 deletions and exon 21 p.L858R), but were not able to detect all rare and more complex alterations in exons 18–20, including some exon 20 insertions. Future improvements of the EasyPGX^®^ ready EGFR assay may allow for the detection of additional exon 20 insertions, which are targetable with recently approved drugs (e.g., amivantamab, mobocertinib) [30,31].

The present study has several limitations. First, the analysis includes only *EGFR* variants covered by the EasyPGX^®^ ready EGFR assay (exon 18 p.G719X, exon 19 deletions, exon 20 p.T790M and p.S768I, selected exon 20 insertions, exon 21 p.L858R and p.L861Q). Therefore, no statement can be made about the detection of other *EGFR* variants not covered by the assay (e.g., the above-mentioned exon 20 p.C797S variant). Second, the analyzed cases all stem from a single tertiary hospital in Switzerland and the study population therefore consists mainly of Swiss/European, non-Asian patients. As the distribution of some driver gene alterations in NSCLC (including but not limited to *EGFR*) is heavily dependent on the tested population [3], the present results may not be generalizable to other (especially Asian) populations. Third, although a broad range of sample types were included in the study, most of the samples (97.8%) were FFPE material (84.6% biopsies/resections and 13.2% cytological cell blocks). Non-FFPE samples such as cytological specimens are underrepresented in the present study cohort. Fourth and last, only NSCLC samples of the non-squamous subtype were analyzed in the present study. Although *EGFR* variants are relatively rare in lung squamous cell carcinomas [32], they do occur and this may further limit the generalizability of the present results.

## 5. Conclusions

The present study demonstrates that the EasyPGX^®^ ready EGFR assay is a valid approach for the rapid detection of common *EGFR* variants in real-world clinical NSCLC samples. The assay provides reliable results for a wide range of sample types, variant allele frequencies and tumor cell contents as well as for DNA input amounts lower than recommended by the manufacturer, improving sample management in small specimens with limited quantity of nucleic acids.

## Figures and Tables

**Table 1:** Clinico-pathologic and genomic characteristics of the clinical performance cohort.

Characteristic	Number (%)
Age (in years) Mean ± standard deviation Range	69.6 ± 10.1 28–98
Gender Male Female	421 (52.4) 383 (47.6)
Histological type NSCLC, non-squamous	804 (100)
Sample type FFPE, biopsy FFPE, resection Cytology, cell block Cytology, cytological specimens	487 (60.6) 193 (24.0) 106 (13.2) 18 (2.2)
*EGFR* mutation status (NGS-based) Positive Negative No valid result	89 (11.1) 713 (88.6) 2 (0.3)
*EGFR* variants (NGS-based) Exon 19 deletion p.L858R Exon 20 insertion p.G719X p.L861Q p.T790M ^a^ Total	45 (49.4) 24 (26.4) 9 (9.9) 7 (7.7) 4 (4.4) 2 (2.2) 91 (100)

^a^ Both cases with the p.T790M variant also had a concurrent p.L858R variant.

**Table 2:** Concordance of *EGFR* test results obtained with the EasyPGX^®^ ready EGFR assay and next-generation sequencing (NGS) ^a^.

EasyPGX^®^ ready EGFR assay		**NGS**								
		**Ex19del**	**p.L858R**	**Ex20ins**	**p.G719X**	**p.L861Q**	**p.T790M**	**Negative**	**Not Conclusive**	**Total**
	Ex19del	44								44
	p.L858R		24 ^b^							24
	Ex20ins			5				2		7
	p.G719X				7			1		8
	p.L861Q					4				4
	p.T790M						2 ^b^			2
	Negative			4				672		676
	Not conclusive	1						38	2	41
	Total	45	24	9	7	4	2	713	2	806 ^b^
	Concordance rate (%)	97.8	100.0	55.6	100.0	100.0	100.0	94.3	100.0	

^a^ Green indicates concordant results. Red indicates discordant results. ^b^ Two samples had concurrent p.L858R and p.T790M variants.

**Table 3:** Characteristics of discordant samples between the EasyPGX^®^ ready EGFR assay (qPCR) and NGS-based *EGFR* testing (NGS).

Discordant Sample	qPCR	NGS	DNA Input Category	Sample Type
p.S768_D770dup (exon 20)	negative	positive	high	Cytology, cell block
p.S768_D770dup (exon 20)	negative	positive	intermediate	FFPE resection
p.N771delinsGF (exon 20)	negative	positive	intermediate	FFPE biopsy
p.E746_A750del (exon 19)	negative	positive	low	FFPE biopsy
Exon 20 insertion	positive	negative	low	FFPE biopsy
Exon 20 insertion	positive	negative	intermediate	FFPE biopsy
p.G719X (exon 18)	positive	negative	intermediate	FFPE biopsy

**Table 4:** Failure rate of the EasyPGX^®^ ready EGFR assay according to DNA input.

DNA Input Category	Number (%)	Failure Rate (%)
High (>15 ng)	276 (34.3)	0
Intermediate (5–15 ng)	273 (34.0)	0.4
Low (<5 ng)	255 (31.7)	15.7
Overall	804 (100)	5.1

**Table 5:** Failure rate of the EasyPGX^®^ ready EGFR assay according to the sample type.

Sample Type	Number (%)	Failure Rate (%)
FFPE biopsy	487 (60.6)	5.7
FFPE resection	193 (24.0)	0.5
Cytology, cell block	106 (13.2)	8.4
Cytology, cytological specimen	18 (2.2)	16.7
Overall	804 (100)	5.1

## Data Availability

The original contributions presented in this study are included in the article/Supplementary Material. Further inquiries can be directed to the corresponding author.

## Supplementary Materials

The following supporting information can be downloaded at: https://www.mdpi.com/article/10.3390/biomedicines13040814/s1; Table S1: EasyPGX^®^ ready EGFR assay results for various DNA inputs of the 5% Multiplex I cfDNA Reference Standard.; Table S2: Verification cohort of the EasyPGX^®^ ready EGFR assay.

## Abbreviations

The following abbreviations are used in this manuscript:

DNA	Deoxyribonucleic acid
EGFR	Epidermal growth factor receptor
FFPE	Formalin-fixed paraffin-embedded
FISH	Fluorescence in situ hybridization
H&E	Hematoxylin and eosin
IHC	Immunohistochemistry
LIMS	Laboratory information management system
NGS	Next generation sequencing
NSCLC	Non-small cell lung cancer
RT-qPCR	Real-time quantitative polymerase chain reaction
TAT	Turnaround time
TKI	Tyrosine kinase inhibitors
VAF	Variant allele frequency
