# Supplementary material for: Real-World Performance of the EasyPGX® Ready Epidermal Growth Factor Receptor Assay for Genomic Testing of Non-Small Cell Lung Cancer Samples
Source: Biomedicines. 2025 Mar 28;13(4):814. doi: 10.3390/biomedicines13040814 (PMC12024863; doi:10.3390/biomedicines13040814)
Supplement: Supplementary file 1 [file biomedicines-13-00814-s001.zip › biomedicines-3506106-supplementary.pdf]

Supplementary Material

Table S1: EasyPGX® ready EGFR assay results for various DNA inputs of the 5% Multiplex I cfDNA Reference Standard.

			DNA input/well		
**Reference material variant**	**VAF (%)**	**Assay tube**	**3ng**	**2ng**	**1ng**
p.G719S	5	p.G719X	Positive	Positive	Failed
p.E746_A750del	5	Ex19del	Positive	Positive	Failed
p.M766_A767insASV	5	Ex20ins	Positive	Positive	Failed
p.T790M	5	p.T790M	Positive	Positive	Failed
p.L858R	5	p.L858R	Positive	Positive	Failed

Ex19del, exon 19 deletion. Ex20ins, exon 20 insertion. VAF, variant allele frequency.

Table S2: Verification cohort of the EasyPGX® ready EGFR assay.

Sample	Sample type	Tumor cell content (%)	EGFR variant (NGS)	VAF (%)	DNA input/ well (ng)	EasyPGX® ready EGFR assay
1	Cell block	30	p.L747_T751del	12.2	30.0	Exon 19 deletion
2	Rx FFPE	70	p.E746_A750del<br>p.T790M	58.6<br>22.3	30.0	Exon 19 deletion<br>p.T790M
3	Cell block	60	p.A767_V769dup	49.6	20.0	Exon 20 insertion
4	Cell block	60	p.L861Q	51.6	20.0	p.L861Q
5	Rx FFPE	50	No variant	N/A	20.0	Negative
6	Rx FFPE	80	No variant	N/A	20.0	Negative
7	Rx FFPE	40	No variant	N/A	30.0	Negative
8	Bx FFPE	60	p.L858R<br>p.T790M	75.7<br>32.2	15.0*	p.L858R<br>p.T790M
9	Rx FFPE	80	p.G719C	44.4	5.0	p.G719X
10	Bx FFPE	80	p.G719S<br>p.768I	56.0<br>64.9	1.5	p.G719X<br>p.S768I
11	Cell block	30	p.E746_A750del	20.6	1.5	Exon 19 deletion
12	Bx FFEE	60	p.E746_A750del	24.6	5.0	Exon 19 deletion
13	Smear	40	p.E746_A750del	70.9	5.0	Exon 19 deletion
14	Bx FFPE	80	p.E746_A750del	47.9	10.0	Exon 19 deletion
15	Bx FFPE	80	p.L747_T751del<br>p.T790M	64.2<br>10.8	5.0	Exon 19 deletion<br>p.T790M
16	Rx FFPE	80	p.S768_D770dup	66.5	10.0	Exon 20 insertion
17	Bx FFPE	30	p.L858R	3.9	5.0	p.L858R
18	Rx FFPE	60	p.L858R	55.6	10.0	p.L858R
19	Bx FFPE	60	p.L858R	57.6	5.0	p.L858R
20	Cell block	70	p.L858R	24.5	2.0	p.L858R

* For sample 8, lower DNA inputs (5.0, 2.0, 1.0ng) were also tested and yielded valid results.
Rx, resection. Bx, biopsy. FFPE, formalin-fixed paraffin-embedded. NGS, next generation sequencing. VAF, variant allele frequency. N/A, not available.
